# From multi-target mechanisms to clinical translation: the analgesic potential of quercetin

**DOI:** 10.3389/fphar.2026.1780749

**Published:** 2026-03-19

**Authors:** Fuquan Wang, Yang Yang, Wen Wang, Bifa Fan

**Affiliations:** Department of Pain Medicine, China-Japan Friendship Hospital, Beijing, China

**Keywords:** analgesia, multi-target therapy, neuroinflammation, neuropathic pain, quercetin

## Abstract

As a major global health challenge, pain often eludes optimal management by current therapies due to limitations like side effects and inadequate efficacy in certain subtypes. Quercetin, a natural flavonoid, has emerged as a promising broad-spectrum analgesic, demonstrating efficacy in diverse pain models including inflammatory, neuropathic, and cancer-related pain. This review systematically delineates the broad-spectrum analgesic efficacy of quercetin across diverse pain conditions, including inflammatory, neuropathic, cancer, and postoperative pain. We comprehensively dissect its sophisticated molecular mechanisms, encompassing the suppression of neuroinflammation via modulating glial cell activation and polarization, the mitigation of oxidative stress and ferroptosis, the regulation of key ion channels and neuroreceptors, and the restoration of neural pathway homeostasis. Furthermore, we discuss innovative drug delivery strategies designed to overcome quercetin’s pharmacokinetic limitations and enhance its therapeutic potential. Despite promising preclinical results, challenges in clinical translation, particularly regarding bioavailability and the need for robust clinical validation, are critically examined. By integrating the latest preclinical and clinical evidence and providing a pain-centric, mechanism-driven analytical framework, this review addresses the critical gap in dedicated research on quercetin’s analgesic properties and offers a novel theoretical basis for the rational development of quercetin-based therapeutic strategies for clinical pain management. This study consolidates evidence on quercetin’s analgesic pharmacology and translational potential, concluding that it represents a viable multi-target candidate, with future research poised to optimize its formulations and validate clinical efficacy for pain management.

## 1 Introduction

As a complex physiological and psychological phenomenon, pain not only causes physical discomfort but also may lead to emotional disorders and a significant decline in quality of life due to the long-term activation of the neuro-immune-endocrine network (Li M. et al., 2025; Rosner et al., 2023; Manzar et al., 2025). In severe cases, it can even result in the loss of labor capacity and impairment of social functions, making it a major global public health challenge (Ren and Dubner, 2010; Jang and Garraway, 2024).

Epidemiological surveys indicate that the global prevalence of chronic pain among adults is approximately 27.5% (Zimmer et al., 2022). Pain is generally classified into acute and chronic categories: acute pain represents a physiological protective response triggered by tissue damage or potential injury, characterized by identifiable etiology and transient duration, with resolution following tissue repair; in contrast, chronic pain is defined as pain persisting beyond 3 months or exceeding the normal tissue healing period, representing a pathological state independent of the initial injury (Raja et al., 2020; Treede et al., 2019). The core pathological mechanisms underlying chronic pain include persistent neuroinflammation, oxidative stress (Teixei et al., 2020), neuronal dysfunction, and peripheral/central sensitization—these interconnected mechanisms form a vicious cycle that perpetuates the persistence of chronic pain.

Currently, clinical pain management mainly relies on opioids, non-steroidal anti-inflammatory drugs (NSAIDs), and antiepileptic drugs; however, these therapies have obvious limitations. For instance, opioids are prone to causing drug dependence and tolerance, while long-term use of NSAIDs may induce gastrointestinal and cardiovascular risks (Garegnani et al., 2025). In addition, most existing drugs have limited efficacy in subtypes such as neuropathic and central pain (Jain, 2008; Thouaye and Yalcin, 2023). Therefore, the development of novel strategies that are safe, effective, and have multi-target regulatory potential is of urgent clinical need.

Quercetin is a natural flavonoid compound widely present in plants, which has become a research hotspot in the field of pain due to its diverse biological activities (Pestana et al., 2024; Jin et al., 2023). It exhibits significant anti-inflammatory (Takeda et al., 2024), antioxidant (Wei et al., 2025), anti-apoptotic (Hu et al., 2019), and neuroprotective effects (Saikia et al., 2024). Notably, quercetin has been the subject of numerous comprehensive reviews, which have extensively elaborated on its general anti-inflammatory potential, therapeutic effects against acne vulgaris, antioxidant capacity, and broad-spectrum protective roles in cardiovascular, metabolic, and oncological diseases as well as overall human health. However, these studies either only briefly mention its analgesic effects as a secondary biological activity or lack a systematic, pain-centric dissection of its mechanisms and translational value. The molecular structure of quercetin endows it with multi-target binding capacity, enabling it to exert analgesic effects by regulating inflammatory pathways, oxidative stress balance, ion channel functions, and cell communication networks. Importantly, quercetin has high safety and mild side effects in long-term application, which forms a sharp contrast with traditional analgesic drugs (Andres et al., 2018).

In recent years, a growing body of studies has confirmed that quercetin exerts potential analgesic effects in various preclinical pain models, while clinical evidence remains limited for specific pain types including rheumatoid arthritis and acute postoperative pain. Its mechanism of action is complex with a wide range of targets, such as inhibiting neuroinflammatory responses, regulating glial cell phenotypic transformation, and suppressing neural cell apoptosis. Collectively, quercetin has demonstrated promising application prospects and potential in pain management.

Against the backdrop of existing quercetin reviews that focus on non-pain-related therapeutic potentials, the core significance of the present review lies in its pain-specialized, systematic integration of current research evidence—filling the gap of a dedicated, mechanism-driven analysis of quercetin’s analgesic properties. Although existing studies have provided compelling evidence for the analgesic effect of quercetin, most tend to focus on a single mechanism or specific pain model, lacking systematic integration and refinement of the quercetin-mediated analgesic network. The functional differences of quercetin in different pain types, the crosstalk between its targets, and its clinical translation potential still require in-depth elaboration. This review aims to systematically integrate existing research evidence and comprehensively dissect the molecular analgesic mechanisms and core targets of quercetin from multiple dimensions, including inflammatory pathway regulation, oxidative stress and ferroptosis intervention, ion channel and neuroreceptor modulation, nerve pathway and apoptosis balance, and glial cell phenotypic polarization. Additionally, it will summarize the latest progress in the optimization of its drug delivery systems and clinical translation. By doing so, this review will clarify the commonalities and specificities of quercetin’s actions in different pain subtypes, providing a solid theoretical basis for subsequent targeted drug development, combination therapy strategy formulation, and clinical application, thereby promoting the standardized application of natural active ingredients in pain management.

## 2 Literature search strategy

A systematic literature search was conducted in PubMed, Web of Science, Embase, Cochrane Library, Scopus and CNKI from January 2000 to October 2025 to identify studies on quercetin’s analgesic effects. The search combined MeSH terms (Quercetin, Flavonols, Pain, Neuropathic Pain, Analgesia, etc.) and free text keywords (quercetin derivatives, inflammatory pain, cancer pain, etc.) with Boolean operators (And/or/not). Only full-text original research (*in vitro*/*in vivo*/clinical) and high-quality reviews in English/Chinese focusing on quercetin’s analgesic mechanisms, efficacy or clinical translation were included; non-pain-related studies, conference abstracts and duplicate publications were excluded.

## 3 Broad-spectrum analgesic effect of quercetin

Quercetin exerts broad-spectrum analgesic potential in various pain models, with its effects manifested in two aspects: direct analgesic effects by inhibiting nociceptive signal transduction and reducing neuronal excitability, and indirect modulation of pain pathophysiology by inhibiting persistent inflammation and oxidative stress, reversing neural sensitization, and protecting nerve tissue integrity (Liu et al., 2022; Carullo et al., 2017) (As shown in Table 1).

**TABLE 1:** Summary of quercetin-mediated analgesic effects and verified molecular mechanisms in different pain models and clinical diseases.

Pain type/Clinical disease	Experimental model/Clinical population	Key mechanisms verified	References
Inflammatory pain - acute	Formalin-induced acute inflammation	Downregulation of IL-1β, IL-2, TNF-α in spinal cord; inhibition of spinal microglial (Iba1+) and astrocyte (GFAP+) activation	Patel et al. (2025)
Inflammatory pain - rheumatoid arthritis (RA)	Collagen-induced arthritis; zymosan-induced arthritis; female RA patients	Inhibition of NF-κB/JAK-STAT pathways; reduced PGE2/NO production; suppressed mast cell activation and inflammatory mediator release	Haleagrahara et al. (2017), Guazelli et al. (2018), Javadi et al. (2017), El-Said et al. (2022)
Peripheral nerve injury	Chronic constriction injury; spinal nerve ligation; brachial plexus avulsion	Inhibition of microglial/astrocytic/satellite glial cell activation; downregulation of pro-inflammatory cytokines; attenuation of oxidative stress	Muto et al. (2018), Huang et al. (2023), Ye et al. (2021)
Neuropathic pain - diabetic neuropathy	STZ-induced diabetic neuropathic pain	Scavenged ROS; inhibited oxidative stress-mediated MAPK pathway activation; suppressed neuronal apoptosis	Saikia et al. (2024), Chis et al. (2017)
Neuropathic pain - chemotherapy-induced peripheral neuropathy	Paclitaxel-induced; cisplatin-induced (PC12 cell model); oxaliplatin-induced	Antioxidant/anti-inflammatory effects; stabilized mast cells; regulated satellite glial cell activity; inhibited ROS-mediated neuronal damage	Gao et al. (2016), Unay and Bilgin (2023), Azevedo et al. (2013)
Central post-spinal cord injury	SCI model	Targeted Ccl4-Ccr5 axis; inhibited CYP1B1 expression and microglial ferroptosis; reduced spinal cord apoptosis and pro-inflammatory cytokine release	Li L. et al. (2025), Zhou et al. (2026), Zhang et al. (2025)
Cancer pain	Breast cancer pain; bone cancer pain	Downregulated HIF-1α/VEGF-A pathway; inhibited PAR2/TRPV1 signaling; reduced pro-inflammatory cytokine release in tumor microenvironment	Calixto-Campos et al. (2015), Li Z. et al. (2018), Cao et al. (2025)
Migraine	Nitroglycerin-induced migraine	Inhibited oxidative stress and inflammatory mediator release; regulated neurotransmitter release and nerve pathway sensitivity	Foudah et al. (2022)
Postoperative pain	Cesarean section patients	Adjuvant analgesic effect (specific clinical mechanism to be further verified)	Mohamed et al. (2025)

### 3.1 Inflammatory pain

Quercetin has exhibited consistent analgesic efficacy across a broad spectrum of acute and chronic inflammatory pain models. In the formalin-induced acute inflammation model (Rat cervical spinal cord hemi-contusion model), quercetin effectively reduced formalin-evoked paw-licking behavior in rats in a dose-dependent manner. This behavior is typically divided into two phases: Phase I (0–7 min), representing acute neuropathic pain, and Phase II (15–60 min), corresponding to inflammatory pain. Notably, the ameliorative effect of quercetin was more pronounced in mitigating Phase II inflammatory pain (Jabbari et al., 2025).

In clinically more relevant chronic arthritis models, such as zymosan-or collagen-induced arthritis (CIA) (mice model), quercetin administration not only significantly alleviated paw edema in mice and shortened the duration of the edema’s severe phase but also exerted long-term beneficial effects: it markedly improved joint function, reduced arthritis scores, and suppressed the infiltration of inflammatory cells in synovial tissue (Haleagrahara et al., 2017; Guazelli et al., 2018). A double-blind, randomized controlled clinical trial further confirmed that quercetin exerts a significant adjuvant therapeutic effect in female patients with rheumatoid arthritis (RA). Specifically, in terms of clinical symptoms, quercetin supplementation significantly shortened the duration of morning stiffness, reduced the severity of morning pain and post-activity pain, and notably decreased the tender joint count (TJC) compared with baseline. Regarding disease activity and functional status, the disease activity score (DAS) was substantially reduced, and the proportion of patients in the active phase of the disease was significantly decreased. Additionally, the plasma level of high-sensitivity tumor necrosis factor-α (hs-TNF-α) was markedly lowered (Randomized controlled trial) (Javadi et al., 2017). This clinical study is a single-center RCT with a moderate sample size, and its results confirm the adjuvant anti-inflammatory and analgesic effect of quercetin in RA patients, with high evidence level for its application in chronic inflammatory pain.

### 3.2 Neuropathic pain

#### 3.2.1 Peripheral nerve injury

In various peripheral nerve injury models, including chronic constriction injury (CCI) of the sciatic nerve, spinal nerve ligation (SNL), and brachial plexus avulsion (BPA), quercetin exerts its therapeutic effects through multiple pathways. Specifically, it can significantly and persistently alleviate mechanical allodynia and thermal hyperalgesia. Concomitantly, quercetin reduces inflammatory cell infiltration at the site of nerve injury, inhibits the excessive activation of satellite glial cells (SNI rat model), microglia (C7 ventral and dorsal nerve root avulsion rat model), and astrocytes (CCI rat model), downregulates the levels of pro-inflammatory cytokines, and mitigates oxidative stress-induced damage (Muto et al., 2018; Du et al., 2023; Huang et al., 2023).

#### 3.2.2 Metabolic neuropathy

In the streptozotocin (STZ)-induced diabetic neuropathic pain model (SNI rat model), quercetin not only significantly reduces blood glucose levels and improves metabolic abnormalities but also inhibits oxidative and nitrosative stress via its potent antioxidant activity. Furthermore, its anti-inflammatory properties can attenuate the inflammatory response in nerve tissues. These combined effects collectively reverse pain-related behaviors such as thermal hyperalgesia in animals, ameliorate histopathological changes including axonal degeneration and myelin sheath damage (Diabetic np rat model), and effectively preserve nerve function (Chis et al., 2017; Kumar et al., 2009).

#### 3.2.3 Chemotherapy-induced peripheral neuropathy (CIPN)

CIPN is a major dose-limiting toxic side effect of chemotherapeutic agents. Studies have shown that quercetin can effectively prevent and treat neuropathic pain induced by various chemotherapeutic drugs, such as paclitaxel (both rat and mouse models) and cisplatin (PC12 cell model), through antioxidant and anti-inflammatory effects (CCI rat model), as well as by stabilizing mast cells and regulating the activity of satellite glial cells (Gao et al., 2016; Ye et al., 2021; Unay and Bilgin, 2023). It significantly ameliorates symptoms such as mechanical allodynia and cold hyperalgesia, while reducing nerve tissue damage and the release of pro-inflammatory cytokines (Gao et al., 2016; Azevedo et al., 2013). Notably, existing studies on quercetin for CIPN are limited to preclinical models, with no clinical evidence available, and the optimal dosage and administration route for clinical application remain to be explored.

#### 3.2.4 Central post-injury pain

Following spinal cord injury (SCI), quercetin not only improves the Basso Mouse Scale score, enhances gait coordination, and promotes motor function recovery in mice but also reduces spinal cord tissue apoptosis and the release of pro-inflammatory cytokines through multiple mechanisms, including targeting the Ccl4-Ccr5 signaling axis, inhibiting CYP1B1 expression, and suppressing microglial ferroptosis (Li L. et al., 2025) (SNI mice model). Consequently, it significantly alleviates neuropathic pain below the level of injury (Li L. et al., 2025; Zhou et al., 2026) (SCI mice model). Similar to CIPN, the research on quercetin for SCI-related central pain is currently in the preclinical stage, and large-sample clinical validation is urgently needed.

### 3.3 Cancer pain and other pain types

Quercetin has demonstrated significant analgesic effects in multiple pain models, including cancer pain, migraine, and postoperative pain. In cancer pain models such as breast cancer and bone cancer, quercetin exerts its effects by downregulating the HIF-1α/VEGF-A pathway (breast cancer mice model), inhibiting PAR2/TRPV1 signaling (bone cancer pain rat model), and reducing the release of pro-inflammatory cytokines. Concomitantly, it suppresses osteoclast activation and bone destruction, thereby markedly ameliorating mechanical and thermal hyperalgesia (Calixto-Campos et al., 2015; Li et al., 2018; Cao et al., 2025). Additionally, quercetin can enhance analgesic efficacy by activating opioid receptors or synergizing with morphine. In the nitroglycerin-induced migraine model (rat model), quercetin alleviated photophobia and tactile allodynia, and improved exploratory and motor behaviors (Foudah et al., 2022). For acute postoperative pain, a prospective, double-blinded, randomized controlled trial (NCT06650891, n = 80) confirmed the safe adjuvant analgesic effect of quercetin in elective cesarean section patients (ASA I–II, spinal anesthesia). Preoperative oral administration of 500 mg quercetin (n = 40) significantly reduced the 2/6/12/24 h postoperative VAS scores, delayed the time to first rescue analgesia (3.9 ± 1.3 vs. 2.73 ± 0.78 h, p < 0.001) and accelerated physical activity initiation (15.2 ± 1.9 vs. 19.03 ± 2.66 h, p < 0.001) compared with the placebo group (n = 40), with standardized concomitant analgesia (bupivacaine/fentanyl spinal anesthesia, diclofenac, acetaminophen, morphine PCA) applied to both groups. No significant differences were observed in 24 h morphine consumption, postoperative adverse reactions or hospital stay between the two groups, and quercetin improved patient satisfaction on postoperative day 2 (p = 0.042) (Mohamed et al., 2025) (RCT). These findings are limited to acute postoperative pain and not extrapolated to chronic or cancer-related pain.

## 4 The molecular mechanisms of Quercetin's analgesic effect

**FIGURE 1:**
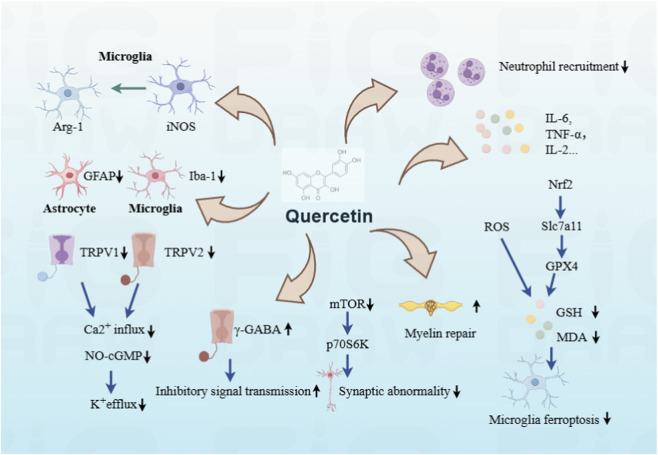
Quercetin alleviates pain through multiple positive mechanisms. Quercetin exerts broad-spectrum pain-relieving effects through various core regulatory pathways: Anti-neuroinflammation: Quercetin inhibits the NF-κB/JAK-STAT signaling pathways to downregulate the secretion of pro-inflammatory cytokines (IL-1β, TNF-α, IL-6) and mediators (PGE2, NO), suppresses neutrophil recruitment in inflammatory foci, and inhibits the abnormal activation of spinal microglia (Iba-1+) and astrocytes (GFAP+); it also modulates microglial phenotypic polarization, downregulating the pro-nociceptive M1 phenotype (iNOS+) and promoting the anti-inflammatory M2 phenotype (Arg-1+), thereby reversing central sensitization. Inhibition of oxidative stress and ferroptosis: Quercetin directly binds to Nrf2 to promote its nuclear translocation, activates the Nrf2/Slc7a11/Gpx4 signaling axis, enhances GSH synthesis, scavenges ROS, reduces MDA accumulation, and specifically inhibits microglial ferroptosis in the spinal cord; protecting nerve myelin sheath integrity and reducing oxidative neuronal damage.Regulation of ion channels and neuroreceptors: Quercetin inhibits the activation of pro-nociceptive ion channels (TRPV1, TRPV2) to block abnormal electrical discharge of sensory neurons and nociceptive signal transduction; it also activates inhibitory GABA/CB1 receptors and modulates the NO-cGMP-K+ pathway to promote K+ efflux and reduce the excitability of primary sensory neurons.

The consistent analgesic activity of quercetin observed in diverse pain scenarios not only breaks the intervention limitation of single pain type but also implies that it may target the core common pathways underlying pain pathogenesis. From the inhibition of pro-inflammatory cytokine release and reversal of neural sensitization in basic experiments to the reduction of pain scores and promotion of functional recovery in clinical studies, the analgesic effect of quercetin exhibits both “broad-spectrum” characteristics and “specific” regulatory features in different pain models. Behind these phenomena lies its precise intervention on key molecular targets in the pain signaling network—encompassing both the cascade regulation of the inflammatory-immune network and the synergistic modulation of multiple core processes such as neurotransmitter release, ion channel function, and redox balance (As shown in Figure 1).

### 4.1 Anti-neuroinflammation

Neuroinflammation represents a core driver of the initiation and persistence of chronic pain, particularly neuropathic pain. Quercetin exerts potent inhibitory effects on neuroinflammation through multiple pathways.

#### 4.1.1 Regulation of inflammatory factors and inflammatory pathways

Quercetin exerts a significant regulatory effect on inflammation-mediated pain, with its core mechanisms focusing on inhibiting the release of pro-inflammatory cytokines and intervening in key inflammatory pathways. In the formalin-induced inflammatory pain model, quercetin downregulates the levels of interleukin-1β (IL-1β), interleukin-2 (IL-2), and tumor necrosis factor-α (TNF-α) in spinal cord tissue, reduces paw licking behavior in rats, and exhibits a more pronounced effect in the inflammation-dominant phase II response (Jabbari et al., 2025). These cytokines serve as crucial mediators that facilitate the phosphorylation of NMDA receptors. This phosphorylation alleviates the magnesium ion blockade of NMDA receptor channels, resulting in an increased influx of calcium ions, which triggers abnormal neuronal firing and excessive excitation (Gruber-Schoffnegger et al., 2013). Concurrently, quercetin significantly inhibits the activation of microglial cells (Iba1-positive) and astrocytes (GFAP-positive) within the spinal dorsal horn. This inhibition disrupts the positive feedback loop whereby pro-inflammatory mediators, such as TNF-α and IL-1β released by glial cells, stimulate neuronal activity, thereby preventing the persistence of a neuroinflammatory state (Patel et al., 2025). For RA, it inhibits synovial cell activation and neutrophil infiltration by targeting the nuclear factor-κB (NF-κB) and Janus kinase-signal transducer and activator of transcription (JAK-STAT) pathways, thereby decreasing the production of prostaglandin E2 (PGE2) and nitric oxide (NO) in the inflammatory microenvironment (RA rat model) (Haleagrahara et al., 2017; El-Said et al., 2022). In osteoarthritis and intervertebral disc degeneration models, quercetin simultaneously suppresses inflammatory responses and the expression of matrix metalloproteinases (MMPs), delays tissue degradation (intervertebral disc degeneration rat model), and alleviates pain-related dysfunction (Li L. et al., 2025; Zhao et al., 2023). Regarding cancer pain, it blocks the central transmission of inflammatory signals by inhibiting macrophage polarization and the release of IL-6 and TNF-α in the tumor microenvironment (Li et al., 2018). In neuroinflammation associated with SCI, quercetin targets the Ccl4–Ccr5 axis, blocks the communication between macrophages and natural killer (NK) cells, inhibits the activation of the downstream NF-κB pathway, and reduces the release of pro-inflammatory cytokines (SCI mice model) (Zhang et al., 2025). In CIA and zymosan-induced arthritis models, quercetin further verifies its anti-inflammatory and analgesic effects by inhibiting mast cell activation and the release of inflammatory mediators (Guazelli et al., 2018; Gao et al., 2016).

#### 4.1.2 Regulation of glial cell activation

In the process of central sensitization in pain, the aberrant activation of microglia and astrocytes constitutes a core pathological link; these 2 cell types collectively drive the establishment and maintenance of the pain state by excessively releasing inflammatory mediators and regulating neuronal signal transduction (Hu et al., 2023; Chen et al., 2018; Yao et al., 2025).

Quercetin can significantly inhibit the excessive activation of glial cells in pain models. In models such as CCI, BPA, and SCI, it downregulates the expression of microglial marker Iba-1 and astrocyte marker GFAP in the spinal dorsal horn and dorsal root ganglion (DRG), while reducing macrophage infiltration and the activation of activation-related signaling pathways (Muto et al., 2018; Huang et al., 2023; Ye et al., 2021). In the CCI model, quercetin inhibits the activation of Iba-1-positive microglia in the spinal dorsal horn, decreases the phosphorylation level of p-JNK, and thereby reduces the release of pro-inflammatory cytokines such as IL-1β and TNF-α(32). For BPA-induced neuropathic pain, it simultaneously suppresses the accumulation of Iba-1-positive microglia, GFAP-positive astrocytes, and CD68-positive macrophages in the C6-C8 segments of the spinal cord, alleviating neuroinflammatory infiltration (Huang et al., 2023). Additionally, in the SNI model, preoperative administration of quercetin can significantly inhibit GFAP expression in DRG satellite glial cells, thereby blocking the development of mechanical hyperalgesia (Muto et al., 2018). In the SCI model, quercetin reduces spinal cord tissue apoptosis and the release of inflammatory factors by targeting and regulating glial cell activation-related signaling pathways (Zhang et al., 2025).

#### 4.1.3 Regulation of glial phenotypic polarization

Quercetin’s regulatory effects on glial cells exhibit multidimensional and targeted characteristics: it not only modulates glial cell activation but also plays an important role in regulating the phenotypic polarization of glial cells (ischemia/reperfusion injury rat model) (Li et al., 2023). In an *in vitro* model of LPS-induced astrocytes, quercetin reverses senescence-related phenotypes, inhibits the release of pro-inflammatory cytokines such as IL-6 and IL-8 in the senescence-associated secretory phenotype (SASP), and downregulates the expression of senescence markers P16 and γ-H2AX (Zhang et al., 2025). In microglial regulation, it suppresses the expression of inducible nitric oxide synthase (iNOS)—a marker of the pro-inflammatory M1 phenotype—and promotes the activation of arginase-1 (Arg-1)—a marker of the anti-inflammatory M2 phenotype, thereby fundamentally reversing the “pro-nociceptive” tendency of the spinal cord microenvironment (Ye et al., 2021). Additionally, quercetin’s regulation of glial cells involves specific molecular targets: for instance, it inhibits satellite glial cell activation by downregulating the co-expression of glial fibrillary acidic protein (GFAP) and P2X4 receptors in the DRG (Muto et al., 2018), or suppresses astrocyte senescence-related inflammatory responses by targeting the clusterin (CLU) gene (Zhang et al., 2025), further enhancing its “anti-nociceptive” effects.

### 4.2 Regulation of oxidative stress and ferroptosis

Oxidative stress and ferroptosis are key pathological processes underlying neuropathic pain and tissue injury-induced pain, and quercetin exerts its effects through multi-targeted interventions. In SCI rats models, quercetin activates the Nrf2/Slc7a11/Gpx4 axis, promotes glutathione (GSH) synthesis, and inhibits malondialdehyde (MDA) accumulation, thereby suppressing microglial ferroptosis, reducing pro-inflammatory cytokine release and neuronal damage (Li L. et al., 2025; Kumar and Vinayak, 2020). Its molecular mechanism involves the direct binding of quercetin to Nrf2 (binding energy: −8.3 kcal/mol), which promotes nuclear translocation and upregulates the expression of downstream antioxidant genes and ferroptosis-inhibitory genes (Kumar and Vinayak, 2020). In diabetic peripheral neuropathy, quercetin scavenges reactive oxygen species (ROS), inhibits oxidative stress-mediated activation of the mitogen-activated protein kinase (MAPK) pathway, improves the integrity of nerve myelin sheaths, and elevates mechanical and thermal pain thresholds (Chis et al., 2017; Gao et al., 2016). In titanium alloy prosthesis-associated arthritis, quercetin alleviates periprosthetic inflammation and pain by regulating oxidative stress pathways (Zhao et al., 2023). In CINP models, quercetin inhibits ROS-mediated neuronal damage and relieves paclitaxel-induced mice model and oxaliplatin-induced mechanical allodynia (Azevedo et al., 2013; Kong et al., 2025).

### 4.3 Regulation of ion channels and neuroreceptors

The precise regulation of ion channels and neuroreceptors by quercetin constitutes a crucial molecular basis for its analgesic effect. In inflammatory and neuropathic pain models, quercetin inhibits the activation of transient receptor potential vanilloid 1 (TRPV1) and TRPV2 channels, reducing neuron sensitization mediated by calcium ion influx, and this effect can be reversed by the TRPV1 antagonist capsazepine (Jabbari et al., 2025). Concurrently, it enhances inhibitory neurotransmission and blocks nociceptive signal transduction by activating γ-aminobutyric acid type A (GABA) receptors and cannabinoid CB1 receptors. In terms of ion channel regulation, quercetin interferes with the NO-cGMP-K channel pathway, promotes K efflux, and decreases the excitability of primary sensory neurons—this mechanism has been verified in both formalin pain and spinal cord contusion models. Additionally, its inhibitory effects on voltage-gated sodium (Nav) channels and hyperpolarization-activated cyclic nucleotide-gated (HCN) channels can block abnormal electrical discharge in neuropathic pain (Azevedo et al., 2013; Liang et al., 2020; Liu et al., 2024). Molecular docking studies have shown that quercetin binds stably to C-C chemokine receptor type 5 (Ccr5) (key amino acid residues: TYR-108 and PHE-109), and reduces pain signal transduction associated with abnormal ion channel activation by inhibiting the Ccl4–Ccr5 axis (Li L. et al., 2025; Zhang et al., 2025).

### 4.4 Regulation of neural pathways and cell apoptosis

Nerve pathway remodeling and imbalanced cell apoptosis are core mechanisms underlying the persistence of chronic pain, and quercetin exerts its effects through multi-dimensional interventions. In SCI-related neuropathic pain, quercetin targets the Ccl4–Ccr5 axis, blocks the communication between macrophages and natural killer (NK) cells, inhibits the activation of the downstream nuclear factor-κB (NF-κB) pathway and spinal cord tissue apoptosis, and significantly improves motor function (Basso Mouse Scale score) and pain thresholds in rats (Li L. et al., 2025; Zhang et al., 2025). In paclitaxel- and oxaliplatin-induced CIPN, it regulates the AMP-activated protein kinase (AMPK)/mitogen-activated protein kinase (MAPK) pathway, inhibits neuronal apoptosis and nerve fiber damage, and relieves mechanical allodynia (Azevedo et al., 2013; Kong et al., 2025). In spinal cord contusion models, quercetin reduces the phosphorylation level of signal transducer and activator of transcription 3 (Stat3), inhibits microglial activation and astrocyte proliferation, and decreases the compression and remodeling of nerve pathways by glial scar (Jabbari et al., 2025). In diabetic peripheral nerve injury, it reduces neuronal apoptosis and improves pain-related behavioral performance by inhibiting synaptic morphological abnormalities mediated by the mammalian target of rapamycin (mTOR)/p70S6K pathway (Chis et al., 2017). In migraine models, quercetin exhibits potential analgesic effects by regulating neurotransmitter release and nerve pathway sensitivity (Foudah et al., 2022).

## 5 Challenges in clinical translation

The low water solubility and bioavailability of quercetin are the core bottlenecks limiting its clinical translation. However, novel drug delivery systems have significantly improved its analgesic efficacy and clinical applicability through precise delivery and controlled-release designs (Tomou et al., 2023). Among nanodrug delivery systems, nanovesicular ethosomal gels, borate complexes, and other formulations can enhance the drug retention capacity and local penetration efficiency at pain sites, thereby prolonging the action duration and increasing the drug concentration at target sites. In diabetic neuropathic pain models, such systems can extend the duration of quercetin’s analgesic effect by 2–3 times and reduce the potential risks associated with systemic exposure (Tomou et al., 2023). The injectable reactive oxygen species (ROS)-responsive methacrylated gelatin hydrogel (MSQ) achieves “on-demand release” and dual pharmacological effects through the synergistic delivery of quercetin and the ROS-scavenging effect of thiol groups. In SCI models, this system can simultaneously inhibit microglial ferroptosis and neuroinflammation via the Nrf2/Slc7a11/Gpx4 pathway. Its analgesic effect is comparable to that of pregabalin, a first-line clinical drug, and it can also promote neural repair and motor function recovery (Li L. et al., 2025; Moghadam et al., 2022). The microneedle patch delivery system effectively avoids hepatic first-pass metabolism through a minimally invasive transdermal delivery method. It exhibits a stable plasma concentration curve and long-lasting analgesic effect in paclitaxel-induced peripheral neuropathic pain models, with the advantages of convenient operation and high patient compliance (Kong et al., 2025; Tomou et al., 2023). In addition, novel carriers such as starch-based photocurable hydrogels can further optimize the *in vitro* release kinetics of quercetin by regulating the network pore structure and hydrophilicity, increasing the cumulative drug release rate to 56.62%, which provides a new option for oral or local administration (Moghadam et al., 2022).

Insufficient verification of efficacy consistency and safety is also an important restrictive factor. Currently, most clinical studies focus on a single pain type, such as RA-related pain, while large-sample, long-term clinical data on refractory pains such as CINP and post-SCI pain are relatively lacking. Although long-term administration of quercetin has not shown obvious abnormalities in liver and kidney functions, novel drug delivery systems may alter the drug metabolism pathway (Pasdar et al., 2020; Liu et al., 2020). Its long-term safety, such as the biocompatibility of carrier materials and the risk of drug accumulation, still needs more clinical data support. In addition, the analgesic effect of quercetin has been mostly confirmed in animal models, and there are few direct comparative studies with first-line clinical analgesic drugs such as pregabalin. Its position in combination therapy, such as adjuvant medication or alternative medication, remains unclear. Moreover, the efficacy differences for different pain subtypes, such as mechanical pain and cold hyperalgesia, have not been clarified, making it difficult to formulate personalized administration plans. All these bring uncertainties to its clinical application.

## 6 Conclusion

As a naturally occurring flavonoid with wide availability and high safety, quercetin holds considerable therapeutic potential in the field of pain management that cannot be ignored. This review elaborates on its broad-spectrum analgesic activity across various pain models. The underlying mechanisms are extremely complex and sophisticated, which cannot be simply summarized by a single target. Instead, quercetin exerts its effects through a multi-dimensional and multi-level regulatory network involving anti-neuroinflammation, anti-oxidative stress, ion channel modulation, neurotransmitter balance, autophagy, epigenetics, and even gut microbiota. This multi-target characteristic endows quercetin with potential advantages over single-target drugs in addressing diseases with complex pathological mechanisms such as chronic pain.

Future research should continue to focus on overcoming the bottleneck of its bioavailability, which is a crucial step for its translation from the laboratory to clinical practice. Meanwhile, more rigorously designed RCTs with larger sample sizes are needed to clarify the effective dosage, safety, and long-term efficacy of quercetin in specific pain populations. With the continuous in-depth exploration of its mechanisms of action, quercetin and its derivatives are expected to become a green and effective novel therapeutic option in the arsenal of future pain management.

## Glossary

AMPK: AMP-activated protein kinase
ASA: American Society of Anesthesiologists
Ccr5: C-C chemokine receptor type 5
CCI: Chronic constriction injury
DAS: Disease activity score
GABA: γ-aminobutyric acid type A
GSH: Glutathione
iNOS: Inducible nitric oxide synthase
IL-6: Interleukin-6
MAPK: Mitogen-activated protein kinase
MMPs: Matrix metalloproteinases
Nav: Voltage-gated sodium
Nrf2: Nuclear factor erythroid 2-related factor 2
NSAIDs: Non-steroidal anti-inflammatory drugs
PGE2: Prostaglandin E2
RCT: Randomized controlled trial
SNI: Spared Nerve Injury
TNF-α: Tumor necrosis factor-α
TRPV2: Transient receptor potential vanilloid 2
Arg-1: Arginase-1
BPA: Brachial plexus avulsion
CIA: Collagen-induced arthritis
CIPN: Chemotherapy-Induced Peripheral Neuropathy
DRG: Dorsal root ganglion
GFAP: Glial fibrillary acidic protein
HCN: Hyperpolarization-activated cyclic nucleotide-gated
IL-1β: Interleukin-1β
JAK-STAT: Janus kinase-signal transducer and activator of transcription
MDA: Malondialdehyde
mTOR: Mammalian target of rapamycin
NF-κB: Nuclear factor-κB
NO: Nitric oxide
PCA: Patient-controlled analgesia
RA: Rheumatoid arthritis
SCI: Spinal cord injury
STZ: Streptozotocin
TRPV1: Transient receptor potential vanilloid 1
VAS: Visual Analogue Scale
